# Long QT Syndrome: Genetics and Future Perspective

**DOI:** 10.1007/s00246-019-02151-x

**Published:** 2019-08-22

**Authors:** Eimear Wallace, Linda Howard, Min Liu, Timothy O’Brien, Deirdre Ward, Sanbing Shen, Terence Prendiville

**Affiliations:** 1 Regenerative Medicine Institute, School of Medicine, National University of Ireland (NUI) Galway, Galway, Ireland; 2 Department of Cardiology, Tallaght University Hospital, Dublin, Ireland; 3 Department of Paediatric Cardiology, Our Lady’s Children’s Hospital Crumlin, Dublin, Ireland

**Keywords:** Long QT syndrome, Arrhythmias, Cardiac, CRISPR–Cas systems, Gene editing, Induced pluripotent stem cells

## Abstract

Long QT syndrome (LQTS) is an inherited primary arrhythmia syndrome that may present with malignant arrhythmia and, rarely, risk of sudden death. The clinical symptoms include palpitations, syncope, and anoxic seizures secondary to ventricular arrhythmia, classically *torsade de pointes*. This predisposition to malignant arrhythmia is from a cardiac ion channelopathy that results in delayed repolarization of the cardiomyocyte action potential. The QT interval on the surface electrocardiogram is a summation of the individual cellular ventricular action potential durations, and hence is a surrogate marker of the abnormal cellular membrane repolarization. Severely affected phenotypes administered current standard of care therapies may not be fully protected from the occurrence of cardiac arrhythmias. There are 17 different subtypes of LQTS associated with monogenic mutations of 15 autosomal dominant genes. It is now possible to model the various LQTS phenotypes through the generation of patient-specific induced pluripotent stem cell-derived cardiomyocytes. RNA interference can silence or suppress the expression of mutant genes. Thus, RNA interference can be a potential therapeutic intervention that may be employed in LQTS to knock out mutant mRNAs which code for the defective proteins. CRISPR/Cas9 is a genome editing technology that offers great potential in elucidating gene function and a potential therapeutic strategy for monogenic disease. Further studies are required to determine whether CRISPR/Cas9 can be employed as an efficacious and safe rescue of the LQTS phenotype. Current progress has raised opportunities to generate in vitro human cardiomyocyte models for drug screening and to explore gene therapy through genome editing.

## Introduction

Long QT syndrome (LQTS), an inherited primary arrhythmia syndrome, demonstrates a prevalence of 1 out of every 2000 healthy live births [1, 2]. This cardiac ion channel repolarization abnormality manifests on the surface electrocardiogram (ECG), as a prolongation of the corrected QT interval, secondary to a delayed repolarization of the cardiomyocyte action potential. This reduction in repolarization reserve places the cardiomyocytes at risk of propagating ventricular arrhythmia from early after depolarizations (EADs) that develop in phase two or three of the action potential [3]. These EADs are a product of the inherent dynamical chaos present in biological systems of cardiomyocytes. When small regions of myocardium develop EADs synchronously, it can trigger focal ventricular tachycardia that, if rapid enough, might result in vortex-like re-entrant excitation of the myocardium, *Torsade de pointes* (TdP). The clinical symptoms of LQTS include palpitations, syncope, and seizures, often due to adrenergic-induced TdP tachycardia [2]. A recessive form of the condition associated with deafness was first described in 1957 (by Jervell and Lange-Nielsen), and an autosomal dominant familial form by Dr. Romano in 1963 and Prof. Conor Ward in 1964. In 1985, Schwartz and Locati were the first to publish on the natural history of the disease and noted a 71% mortality rate in untreated patients from the first syncope [4]. Mortality in the current era for patients with LQTS with appropriate medical therapy is now 0.3% [5].

## Genetics of LQTS

LQTS has been classified into 17 subtypes (see Table 1) based on mutations associated with 15 autosomal dominant genes, LQT1-15 [6, 7].

**Table 1:** Classification of genes responsible for cardiac channelopathies. Adapted from Schwartz et al. [2]

LQTS type	Gene	Mutation frequency among LQTS population (%)	Locus	Protein (functional effect)
Romano–Ward (RWS)				
LQT1	KCNQ1	40–55	11p15.5	K_V_7.1 (↓)
LQT2	KCNH2	30–45	7q35–36	K_V_11.1 (↓)
LQT3	SCN5A	5–10	3p21–24	Na_V_1.5 (↑)
LQT4	ANKB	< 1	4q25–27	Ankyrin B (↓)
LQT5	KCNE1	< 1	21q22.1	MinK (↓)
LQT6	KCNE2	< 1	21q22.1	MiRP1 (↓)
LQT7	KCNJ2	< 1	17q23	Kir2.1 (↓)
LQT8	CACNA1C	< 1	12p13.3	L-type calcium channel (↑)
LQT9	CAV3	< 1	3p25	Caveolin 3 (↓)
LQT10	SCN4B	< 1	11q23.3	Sodium channel-β4 (↓)
LQT11	AKAP9	< 1	7q21–22	Yotiao (↓)
LQT12	SNTA1	< 1	20q11.2	Syntrophin α1 (↓)
LQT13	KCNJ5	< 1	11q24	Kir3.4 (↓)
LQT14	CALM1	< 1	14q32.11	Calmodulin 1 (dysfunctional Ca^2+^ signaling)
LQT15	CALM2	< 1	2p21	Calmodulin 2 (dysfunctional Ca^2+^ signaling)
Jervell and Lange-Nielsen syndrome (JLNS)				
JLN1	KCNQ1	< 1	11p15.5	K_V_7.1 (↓)
JLN2	KCNE1	< 1	21q22.1–22.2	MinK (↓)

Arrows up (↑) or down (↓) showing gain or loss of protein function, respectively

*LQT* long QT, *RWS* Romano–Ward syndrome, *JLNS* Jervell and Lange-Nielsen syndrome

**Fig. 1:**
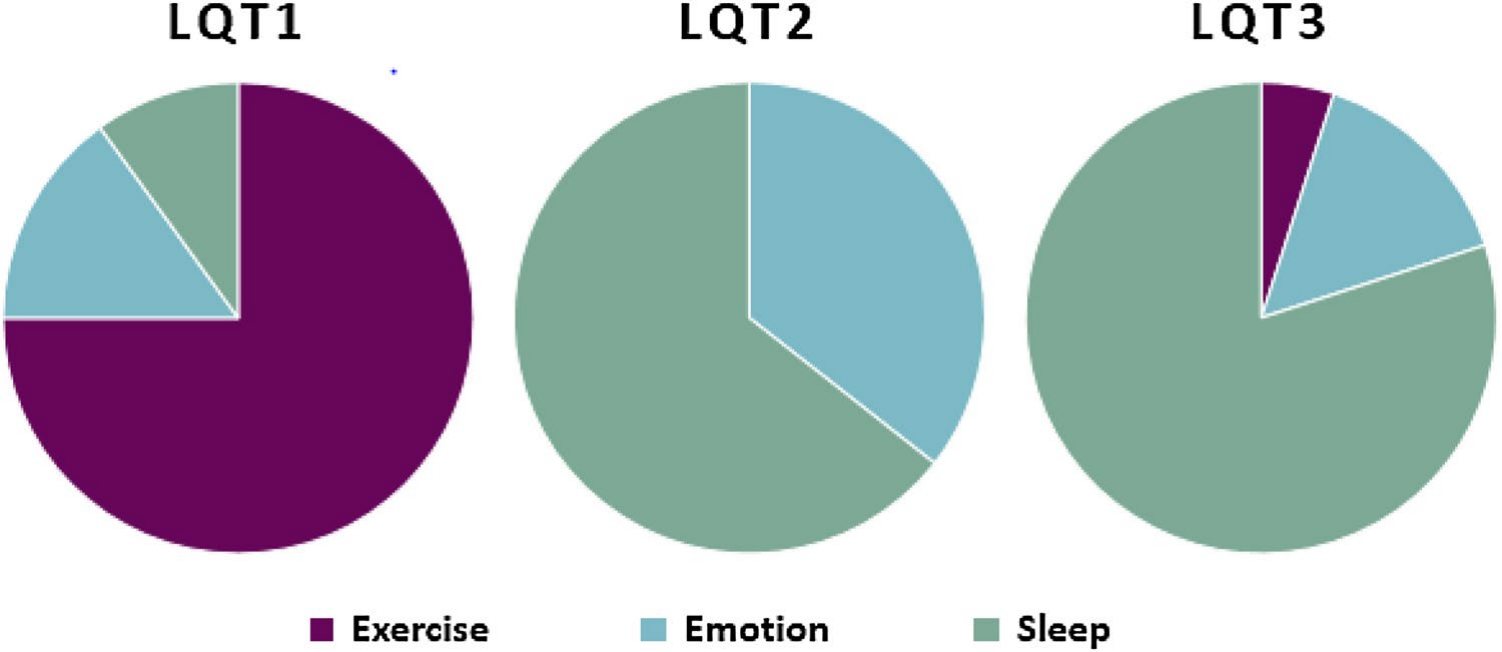
Triggers for cardiac arrhythmias in LQT1, LQT2, and LQT3 by exercise, emotion, and sleep/rest. Adapted from Schwartz et al. [7]

LQT1 the most common subtype affects 30–35% of LQTS individuals and arises from the loss-of-function of *KCNQ1* gene encoding the α-subunit of a voltage-gated potassium channel, K_V_7.1, expressed within the cell membrane of cardiomyocytes. K_V_7.1 mediates a slowly activating delayed rectifier potassium current (*I*_Ks_). K_V_7.1 consists of four α-subunits which co-assemble with *KCNE1* β-subunits to generate the I_Ks_ current. The *KCNQ1* α-subunit has a voltage sensing domain (S1–4), a pore forming domain (S5–6), as well as intracellular N- and C-termini [8]. LQT1 manifests on the surface electrocardiogram as a broad-based and symmetrical T-wave with a prolonged QTc interval [9]. The incidence of life-threatening events is lowest for LQT1 compared to LQT2 or -3 [10]. Βeta-blockers are most effective in LQT1 at preventing breakthrough cardiac events [11]*.* At present, over 600 variants of *KCNQ1* causing LQT1 have been described [12]. The location of a particular LQT1 mutation within the ion channel structure may be directly related to risk of cardiac event. The α-subunit is composed of an N-terminus, six membrane-spanning segments (S1–S6), two cytoplasmic loops (between S2–S3 and S4–S5), and the C-terminus portion. The presence of a mutation in the C-loop structure confers the highest risk for aborted cardiac arrest or sudden death [13]. The inverse correlate of this finding is that there may be a strategy to potentially avoid beta-blockers in low-risk individuals with LQT1 who do not harbor a C-loop mutation although this conclusion is somewhat controversial [14]. As shown in Fig. 1, physical exercise is the primary trigger for syncope or cardiac arrest in LQT1 [2].

LQT2 is the second most common subtype affecting 25–30% of LQTS individuals. hERG (human *Ether-à-go-go*-Related Gene) or *KCNH2* codes for the voltage-gated pore forming α-subunit of the inwardly rectifying potassium channel subunit K_V_11.1. The *KCNH2* α-subunits form a complex with *KCNE2*, a single transmembrane protein homologous to *KCNE1*, to generate the *I*_Kr_ current, intimately involved in membrane repolarization [8]. Heterozygote *KCNH2* mutations exert a dominant-negative effect on wild-type hERG channel-associated *I*_Kr_ currents by impairing trafficking pathways or altered channel kinetics of the resulting co-assembled *hERG* heterotetramers [15]. LQT2 mutations within the *hERG* potassium channel are loss-of-function mutations which reduce *I*_Kr_ amplitudes and thus prolong cardiac repolarization. LQT2 manifests on the surface electrocardiogram as a bifid or notched T-wave that is asymmetrical and of low amplitude [9]. LQT2 is recognized to pose a significant cardiac event risk in the first nine months postpartum [16]. A sudden startle or loud noise and emotional stress are potential triggers for *KCNH2* gene mutation-associated cardiac arrhythmias as illustrated in Fig. 1 [2].

The *SCN5A* gene encodes the α-subunit of the cardiac sodium ion channel Na_V_1.5 that functions as either a monomer or assembles as a dimer in an ion channel complex [17]. The gain-of-function mutations of *SCN5A* disrupt the fast inactivation of the cardiac sodium channels and are associated with LQT3 phenotype, accounting for 5–10% of total LQTS cases [18]. A continuous influx of sodium ions occurs during the plateau phase of the action potential, which delays ventricular repolarization and in turn prolongs the QT interval on the surface ECG [19]. LQT3 may manifest on the surface electrocardiogram as a prolonged isoelectric interval preceding a relatively normal T-wave morphology [9]. It is the subtype of LQTS that is least responsive to beta-blockers and yet is the most lethal [10, 11]. Over 300 *SCN5A* variants are known to be related to LQT3. A wide scale of interacting proteins acting as part of a macromolecular complex regulating function or membrane expression of Na_V_1.5 have been identified [20]. Clinically, LQT3 arrhythmia events are often associated with bradycardia, hence LQT3 patients as depicted in Fig. 1, present with malignant arrhythmias at rest and during sleep due to the strong frequency dependence of this effect [21].

Jervell and Lange-Nielsen syndrome (JLNS), a relatively rare form of LQTS, is an autosomal recessive disorder associated with congenital profound sensorineural hearing loss and usually marked QTc prolongation. JLNS arises from homozygous or compound heterozygous mutations in either *KCNQ1* or *KCNE1*. These genes encode the α and β subunits, respectively, of the potassium ion channel conducting the slow component of the delayed rectifier current [22]. Sporadic cases of JLNS have been reported [2].

An additional six rare forms of LQTS involve ion channels: *KCNE1* (LQT5) as potassium voltage-gated channel subfamily E regulatory subunit 1, *KCNE2* (LQT6) encoding potassium voltage-gated channel subfamily E regulatory subunit 2, *KCNJ2* (LQT7) for inward rectifier potassium channel K_IR_2.1, *CACNA1* (LQT8) for L-type calcium channel subunit, *SCN4B* (LQT10) for sodium channel-β4, and *KCNJ5* (LQT13) for K_IR_3.4 [2, 6]. Note, LQT7, or Andersen–Tawil syndrome, is characterized by the clinical triad of periodic paralysis, ventricular arrhythmias, and prolonged QT interval in association with dysmorphic anatomical features [23].

Three additional rare forms of LQTS involve adapter proteins linking the cell membrane to the cytoskeleton, such as *ANK2* (LQT4) for ankyrin 2, *CAV3* (LQT9) for cavolin 3, and *SNTA1* (LQT12) encoding syntrophin α1 [2, 6].

Other very rare LQTS variants are related to kinase activities, such as *AKAP9* (LQT11) encoding A-kinase anchoring protein 9 binding to regulatory subunit of PKA, *CALM1* (LQT14), and *CALM2* (LQT15) for calmodulin 1 and 2, calcium binding phosphorylase kinase delta [6].

## Diagnosis and Genetic Testing

LQTS is diagnosed by careful clinical and family history including symptoms of syncope, anoxic seizures, or, rarely, palpitations and through targeted investigations including ECGs, 24-h Holter recording analysis, and exercise stress testing (Table 2). Occasionally, provocative drug challenges may play a role in differentiating the diagnoses from other primary arrhythmia syndromes. The ECG of a patient with LQTS characteristically shows QT prolongation when measured appropriately in leads II or V5 using a correction formula for heart rate (QTc). QTc values correlate with risk of life-threatening arrhythmia event with a linear relationship between increasing QTc and increasing risk for all three common genotypes, LQT1, 2, and 3 [24]. It should be noted, however, that there is an overlap with the upper limit of normal QTc values in the background population, hence the importance of the clinical context in making the diagnosis [25]. To assist with collating the variables in making this diagnosis clinically, a scoring system has been devised (Table 3). The scoring system deems a score ≤ 1 as a low probability of LQTS, a score of 1.5–3 as an intermediate probability of LQTS, and a score ≥ 5 as a high probability of LQTS [7]. Exercise stress testing looking for paradoxical prolongation of the QTc with exercise or in recovery forms part of the core diagnostic work-up as does 24-h Holter QToc analysis [26, 27]. In addition to the clinical work-up of a patient suspected of having LQTS, molecular genetics for known LQTS mutations and familial cascade screening of first-degree family members help augment the diagnostic confidence. Genetic screening for LQTS mutations also identifies asymptomatic and phenotype-negative LQTS individuals that might otherwise come to harm from their disease where they are exposed to additional exogenous risk factors including QT-prolonging pharmaceutical drugs [25].

**Table 2:**
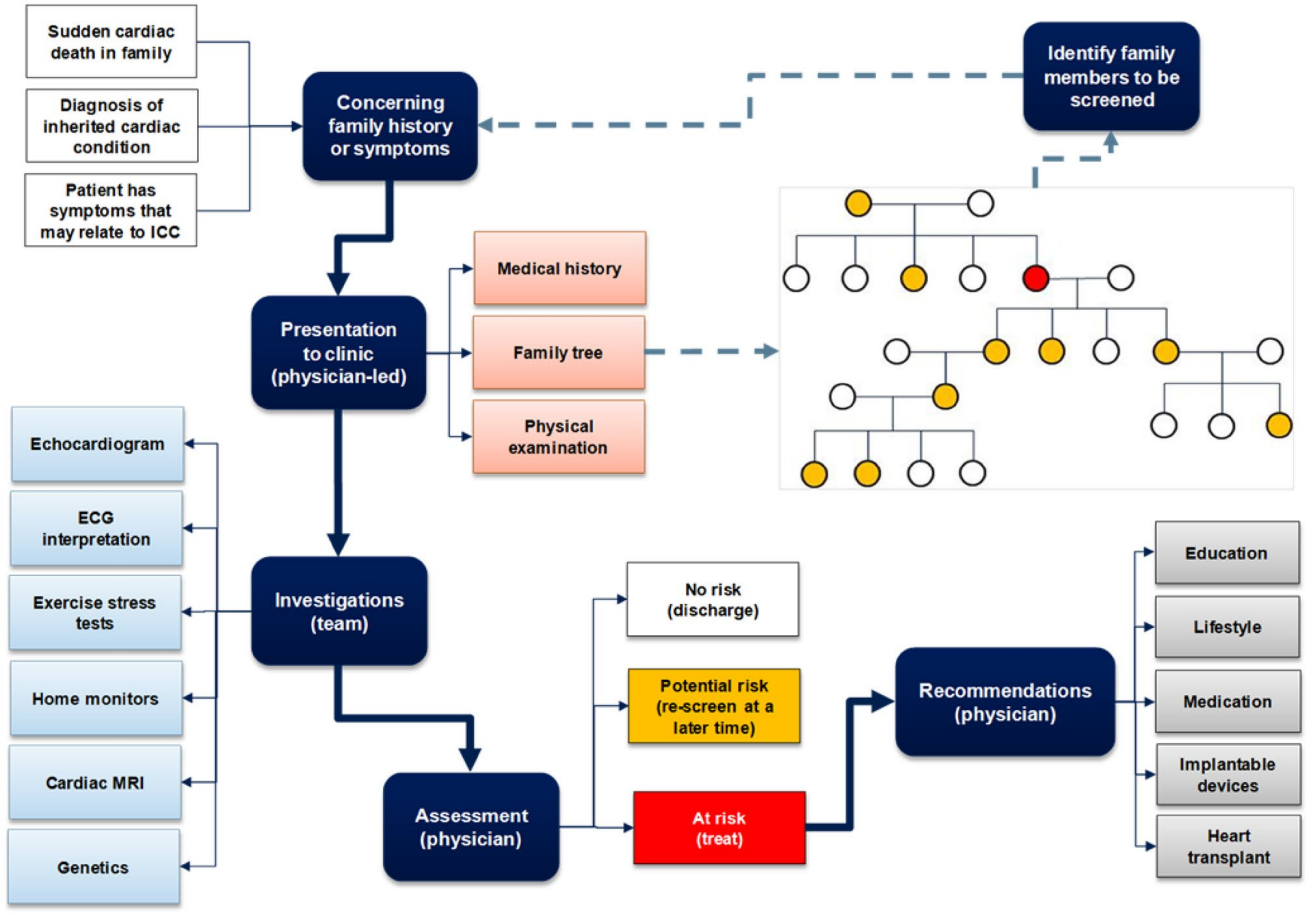
Diagnostic work-up for a patient suspected of harboring a diagnosis of Long QT syndrome

**Table 3:** 1993–2011 LQTS diagnostic criteria—the Schwartz scoring scale. Adapted from Schwartz et al. [7]

	Points
*Electrocardiographic findings*	
A	
QT_C_^a^	
≥ 480 ms	3
460**–**479 ms	2
450–459 ms (male)	1
B	
QT_C_^a^—4th minute of recovery from exercise stress test ≥ 480 ms	1
C	
*Torsade de Pointes*^b^	2
D	
T-wave alternans	1
E	
Notched T-wave in 3 leads^c^	1
F	
Low heart rate for age	0.5
*Clinical history*	
A	
Syncope^b^	
With stress	2
Without stress	1
B	
Congenital deafness	0.5
*Family history*	
A	
Family members with definite LQTS^d^	1
B	
Unexplained sudden cardiac death below age 30 among immediate family members^d^	0.5

^a^QTC calculated by Bazett’s formula where QT_C_ = QT/√PR

^b^Mutually exclusive

^c^Resting heart rate below the 2nd percentile for age

^d^The same family member cannot be counted in A and B

## Standard of Care Therapy

The current standard of care therapy for LQT1, 2, and 3 includes non-cardioselective beta-blockers, preferentially nadolol, that are hypothesized to dampen down adrenergic stimulation of the heart. Beta-blockers are proven to reduce the occurrence of life-threatening ventricular arrhythmias and subsequent risk of sudden death in LQTS patients [24, 28].

In addition to beta-blockers, there are limited data to suggest that LQT2 patients can be prescribed potassium supplements or potassium-sparing diuretics, such as spironolactone, to increase serum potassium levels with a resultant decrease in QTc on ECG [29]. Female patients with LQT2 appear to suffer a greater lifetime risk from cardiac arrhythmias [7]. The 9-month postpartum period sees female patients with LQTS at an increased risk of experiencing a life-threatening event and it is imperative that female patients prioritize beta-blocker therapy in this relatively high-risk period [16].

Recently, the application of a drug developed to assist with ion channel trafficking to the cell membrane in cystic fibrosis has shown promise for rescuing the phenotype of LQTS in patients with trafficking defects in *hERG* [30].

There has been a long-standing concern over the utility of beta-blockade in preventing cardiac arrhythmia events, including sudden death, in patients with LQT3 [31]. Recently, the use of beta-blockade in patients with LQT3 has been shown to be protective [24]. Given the gain-of-function mutation in LQT3, it is mechanistically intuitive that sodium channel blockade would ameliorate risk of arrhythmia. Indeed, mexiletine, a non-selective, voltage-gated sodium channel blocker, is proven to shorten the QTc of patients with LQT3 and significantly reduce the burden of arrhythmic events [24].

LQT4 or Ankyrin B syndrome is known to have an adrenergic trigger with emotional or exertional stress and, intuitively, non-selective beta-blockers may be a therapeutic consideration but there is a lack of evidence for any specific therapies in this rare form of QT prolongation not directly related to ion channelopathy [32].

LQT5 and LQT6 arise from defects to *I*_Ks_ and *I*_Kr_, respectively, and hence are treated with beta-blockers similarly to LQT1 And LQT2 [33].

Patients with LQT7 or Andersen–Tawil syndrome, a *KCNJ2* ion channelopathy encoding the inward rectifier K^+^ channel K_IR_2.1, respond to empiric treatment with flecainide [34]. Potassium supplementation or potassium-sparing diuretics, which increase serum potassium levels, also play a role in therapeutic management [35].

LQT8, or Timothy syndrome, a very rare cause of LQTS, is characterized by risk of severe ventricular arrhythmias associated with a phenotype of severe QTc prolongation, congenital heart defects, syndactyly, facial dysmorphism, and neurodevelopmental delay. Mutations in the CACNA1C cause all reported cases of Timothy Syndrome. These gain-of-function mutations result in increased calcium transport into cardiomyocytes, thereby prolonging QT interval and increasing risk of arrhythmia. The mainstay of therapy is beta-blockade [36].

LQT9 manifests as a repolarization abnormality due to the abnormal structure of the caveolin-3 protein which causes the voltage-gated sodium channel to permit the over-excessive influx of sodium ions. Hence, LQT9 patients are also prescribed sodium channel antagonists such as quinidine to shorten their QTc [37].

Standard of care therapy for the remaining forms of LQTS are limited to the administration of beta-blockers due to the rarity of those subtypes, with limited evidence for efficacy [7].

A minority of LQTS patients continue to be at significant risk of ventricular arrhythmias, cardiac arrest, or sudden unexpected death regardless of medical pharmacotherapeutic efforts. Typically, these patients have a more severe phenotype manifesting as QTc values greater than 500 ms on resting ECG, exercise stress testing, or on Holter QToc analysis [11].

Increasingly, left cardiac sympathetic denervation is considered in patients with breakthrough events, including appropriate implantable cardioverter defibrillator (ICD) shocks for LQTS-related arrhythmias, despite maximal medical therapy [38], or for those who are intolerant of beta-blockers.

With regard to lifestyle modifications for patients with LQTS, all LQTS patients are advised to avoid ingesting QT-prolonging medications. Those with LQT2 are generally advised to avoid low potassium states and to avoid the possibility of being suddenly woken from sleep by a loud noise due to the risk of potentially triggering cardiac arrhythmia. Hence, alarm clocks and telephones that might elicit a startle response should be avoided in the bedroom wherever possible [2].

Recent consensus statement guidelines have become more permissive in exercise participation. Prior to consideration in competitive sports participation, a patient with LQTS should be asymptomatic of their disease on appropriate medical therapy for a period of at least three months; they should continue to avoid QT-prolonging drugs, ensure electrolyte replenishment and avoid dehydration, avoid hyperthermia or training-related heat exhaustion, ensure an automated external defibrillator is available within 5 min of the sports facility, and establish an emergency plan with the appropriate training team [39].

## Induced Pluripotent Stem Cells to Model LQTS In Vitro

**Fig. 2:**
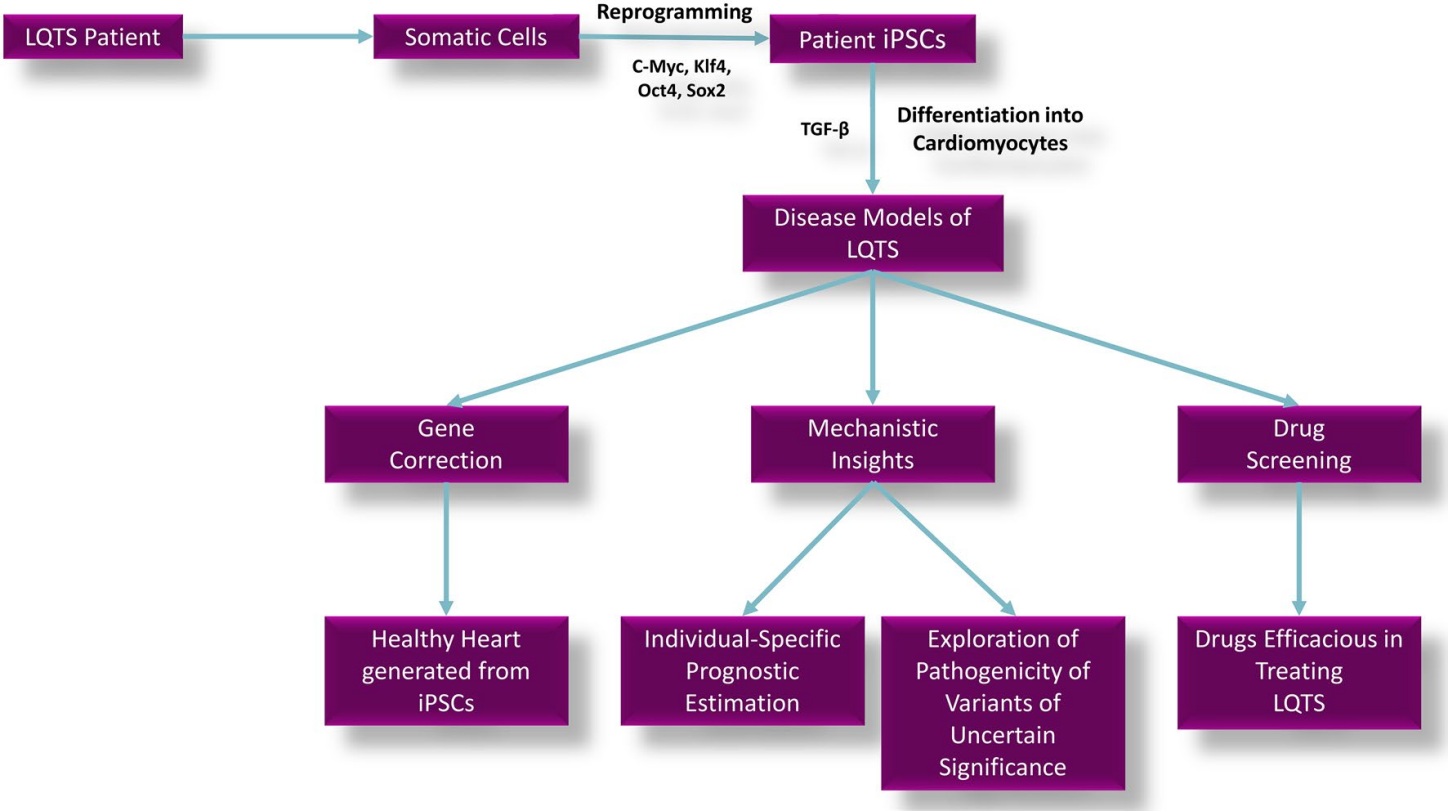
Overview of the applications of iPSCs in LQTS research. C-Myc, Klf4, Oct4, Sox2 are transcription factors used to reprogram patient somatic cells into iPSCs. TGF-β is a growth factor used to differentiate iPSCs into cardiomyocytes. iPSCs, induced pluripotent stem cells. Adapted from Li et al. [68]

The ability to generate induced pluripotent stem cells (iPSC) has revolutionized the field of cardiovascular research, allowing functional analysis of patient-specific cardiac tissue in vitro (see Fig. 2). Historically, mechanisms of cardiac ion channelopathy were based on isolated heterologous expression of a component of the ion channel in a non-cardiac cell as human cardiomyocytes are not amenable to harvesting or culture in vitro [40]. In contrast to this, stem cell technology allows investigators to produce an immortalized, patient-specific iPSC line from skin fibroblasts obtained from a family of interest and to differentiate these iPSCs to a beating syncytium of mature ventricular cardiomyocytes that faithfully replicate both intracellular and intercellular electrical coupling seen in disease states.

Human skin biopsies or white blood cells in a phlebotomy sample act as a source of fibroblasts which can be re-programmed to produce human iPSCs (hiPSCs) and subsequently differentiated along a cardiomyocyte lineage.

The early production of iPSCs relied on integration of retro- or lentiviruses into the cell genome to activate the foetal gene program. These vectors, however, randomly integrated into the native genome, potentially disrupting endogenous gene(s) between experimental iPSC lines. More recently, Sendai-virus has been utilized as non-integrating vector for foetal gene delivery [41]. The process from fibroblasts to iPSCs is elaborate, labor intensive, and costly and requires at least three months of laboratory time from fibroblast harvest to generation of iPSC line [41, 42].

The iPSCs can be differentiated into cardiomyocytes with atrial, ventricular, and pacemaker characteristics. The use of either the whole cell patch-clamp technique or intracellular recording with microelectrodes demonstrates that these iPSC-derived cardiomyocytes (CMs) faithfully generate action potential characteristics [41]. Multielectrode arrays have also been employed in iPSC-derived CM to record their local field potential (‘cellular ECG’) [43]. The duration of the obtained field potentials correlate with the duration of the QT interval on the surface ECG [44].

### Modeling of LQTS

#### LQT1

Moretti et al*.* were the first to publish a fibroblast-derived iPSC model for LQTS, using cells from healthy controls and two asymptomatic patients carrying a *KCNQ1*-G569A gene mutation [45]. Both atrial-like and ventricular-like cardiomyocytes from the LQT1 patient iPSCs demonstrated significantly prolonged action potentials compared to the healthy control cells. The pro-arrhythmogenic effect of isoproterenol in the iPSC-derived CM was ameliorated by beta-blockers, replicating the clinical features of LQTS in vitro [45, 46]. Numerous authors since have generated iPSC-derived CM models of autosomal dominant, recessive, and compound heterozygous LQT1 with subsequent phenotype analysis and drug response testing [47-51].

#### LQT2

Itzhaki et al*.* were among the first groups to report an iPSC model of LQT2. Patient-specific iPSC-derived CMs were developed from a LQT2 patient with an A614 missense mutation that recapitulated a prolonged action potential on patch clamping. In both atrial-like and ventricular-like LQT2 iPSC-derived CM, EADs were detected, and E-4031, a specific pharmacological blocker of the hERG channel, prolonged the action potential duration in both control and patent-specific iPSC-derived CMs [52].

Matsa et al*.* produced iPSC-derived CM from a clinically symptomatic patient and their clinically asymptomatic mother harboring the *KCNH2* G1681A (A561V) mutation. Despite both related family members possessing the same mutation, their iPSC-derived CM displayed varying pathogenicity of phenotype, replicating the in vivo clinical analysis of these two patients mirroring the concept of disease penetrance in vitro [53].

Bellni et al*.* similarly studied the concept of disease penetrance in vitro through the development of isogenic cases and controls with a specific *KCNH2* mutation, A2987T (N996I), that affects intracellular protein trafficking and results in a reduction in repolarizing potassium current in a disease mechanism of haploinsufficiency. By entirely controlling for genetic background in an isogenic experimental design, the authors definitively modeled the genotype–phenotype correlation for this particular mutation whose disease mechanism had been unclear [54].

An interesting observation was noted by Lahti et al. comparing the differences in AP duration, field potential duration (FPD), and QTc on ECG between LQT2 cases and controls. Although a large signal difference was often noted on single cell patch-clamp action potential, the differences between disease and control iPSC-derived CM were more modest by microelectrode arrays, similar to the surface electrocardiogram in such patients. They postulated a ‘rescue’ mechanism that cell-to-cell contacts in the syncytium result in compensatory temporizing responses and a tendency to protect the repolarization system from major deviation in normal physiological parameters [55].

Garg et al. have demonstrated the pathogenicity of a *KCNH2* mutation previously classified as a ‘variant of uncertain significance’ in vitro using CRISPR/Cas9 gene editing to rescue the disease phenotype and to generate an isogenic control [56].

#### LQT3

LQT3 was first modeled in murine iPSCs by Malan et al*.* [57]. The same group subsequently demonstrated the genotype-specific response of mexiletine in shortening the action potential duration and lessening EADs in iPSC-derived CMs from a patient with LQT3 [21].

Veerman et al. studied the duration of iPSC-derived CM culture demonstrating a bearing on the isoform of sodium channel protein expression that potentially would have an effect on replicating a disease state, highlighting one of the challenges of working with ‘physiologically immature’ iPSC CM models [58].

## CRISPR/Cas9 and Its Potential in Disease Modeling and as a Therapeutic in LQTS

CRISPR/Cas9 is an abbreviation for clustered regularly interspaced short palindromic repeats (CRISPR) and CRISPR-associated protein 9 (Cas9). The CRISPR/Cas9 is a highly accurate and efficient genome editing technique, which is faster and cheaper than other preceding gene editing technologies [59]. CRISPR/Cas9 is based on the mechanism of bacterial ‘memory’ for previously encountered viruses [60]. Researchers have manipulated this bacterial defense mechanism to produce this novel genome editing technique. The CRISPR component can be manipulated to match a disease-specific mutation via a short guide RNA molecule of approximately 20 bases, which corresponds to a specific DNA sequence in the genome for Cas9 binding [59]. The double-stranded break (DSB) in DNA inflicted by Cas9 undergoes either non-homologous end joining (NHEJ) or homology-directed repair (HDR) if a closely matching DNA molecular template is provided. Thus, CRISPR/Cas9 can be used to manipulate the DNA repair machinery of a cell to add or delete genetic material, or replace the existing DNA segment with a customized DNA sequence [61].

CRISPR/Cas9 can generate isogenic mutant lines from control iPSCs, or genetically corrected iPSCs from mutant lines, thus eliminating epigenetic differences or unknown genetic modifiers which may introduce phenotype variability in studying disease-causing mutations in LQTS. Therefore, quantifiable phenotypes can be specifically attributed to their mutations and the mechanisms by which mutations cause diseases such as LQTS can be identified. CRISPR/Cas9 can be employed in LQTS modeling to generate ideal controls for the patient-derived iPSCs by correcting the known mutations, hence negating the influence of the genetic background. CRISPR/Cas9 results in such precise genome editing that off-target mutagenesis is minimal and with further development the hope is that it will offer a pathway to potential curative treatment for monogenic diseases such as LQTS.

To successfully treat LQTS, a high percentage of cardiomyocytes need to be edited by CRISPR technology. Hence, viral delivery of guide RNA should be employed to obtain maximum levels of Cas9 delivery to cardiomyocytes. However, viral delivery of Cas9 frequently results in mosaic gene disruption; i.e., only a subset of cardiomyocytes are edited. Adeno-associated virus (AAV9), considered as a hugely efficient viral vector, delivered Cas9 to 60–70% of cardiomyocytes. However, less than 15% of cardiomyocytes were effectively edited by this once off delivery of Cas9. This inefficient editing of cardiomyocytes is likely due the reduced chromatin assembly in post-mitotic cells. Hence, inefficient gene disruption is the main limitation of CRISPR/Cas9 [62].

Another limitation of CRISPR/Cas9 is its apparent ability to evoke an immune response in animals with a functioning immune system. Recent studies have suggested that administration of Cas9 to animal models of diseases evokes an immune response which does not respond to immunosuppressants. The infiltration of immune cells and, in particular, Cas9-specific antibodies, may destroy the edited cells. Hence, immune responses to CRISPR/Cas9 may prove a major limitation for in vivo studies [63]

## RNAi and Its Therapeutic Potential in LQTS

RNA interference (RNAi)-based therapeutics may prove an effective adjunct to standard of care therapy in LQTS. RNAi silences protein production in a sequence-specific manner, hence their potential role in LQTS dominant-negative mutations.

Lu et al*.* demonstrated the effectiveness of RNAi in the treatment of a LQT2 dominant-negative mutation of E637K-hERG. Lu et al*.* rectified this trafficking-deficient mutation using siRNA targeted for the specific E637K-hERG mutant [64]. This technique attenuated the effects of the E637K-hERG mutation so that the kinetic properties of the mutated channel increased to levels seen with the normal hERG channel [64, 65]. However, it remains to be established if this technology works in vivo and if one copy of the normal gene is sufficient to maintain normal function in vivo.

Matsa et al*.* combined the use of RNAi-based therapeutics and iPSCs in the treatment of LQT2 [66]. This allele-specific RNAi inhibited the production of functional levels of mutant proteins. RNAi exerts its functional effects through conversion of the dominant-negative effect to haploinsufficiency.

Studies have demonstrated the potential RNAi offers for the treatment of LQTS and its ability to be patient-specific in cultured cells. RNAi should exert functional effects in vivo provided it efficiently inhibits the production of mutant proteins while leaving wild-type RNA unaffected [66].

## Conclusion

LQTS is a rare inherited cardiac condition associated with risk of malignant ventricular arrhythmias. Diagnosis of LQTS can be challenging as an individual’s first presentation may be with sudden death. QT interval prolongation on resting ECG is pathognomonic of disease, but up to one-third of mutation carriers may have normal QT intervals on resting ECGs. Genetic testing for cardiac ion channelopathy mutations is now an essential component to diagnostic evaluation and familial cascade screening.

The three major subtypes of LQTS are LQT1, LQT2, and LQT3, caused by mutations in the ion channel genes *KCNQ1*, *KCNH2*, and *SCN5A,* respectively. Together, they account for approximately 90% of all genotype-positive cases.

Current standard of care therapy for LQTS is the prescription of non-cardioselective beta-blockers. ICDs and/or left cervical sympathetic denervation may be employed in cases where therapeutic doses of appropriate pharmacotherapy are ineffective in preventing subsequent cardiac events. LQTS individuals are counseled to avoid arrhythmogenic triggers such as QT-prolonging medications, and to exercise with caution following international guideline recommendations. Increasingly, a more nuanced understanding of LQTS pathophysiology is leading towards genotype-specific therapies such as the role of mexiletine for a subset of patients with LQT3 [67]. At present, standard of care therapies do not entirely protect patients from all cardiac events and a small minority remain at clinical risk of sudden death.

The ability to generate cardiomyocytes from skin biopsies using iPSC technology has dramatically enhanced our capacity to model LQTS phenotypes in vitro*.* Patient-specific iPSC-derived CMs offer the potential to understand individual disease processes and to potentially target in vitro models of genotype rescue using CRISPR/Cas9 genome editing technology. Recent advances in iPSC, RNAi, and CRISPR/Cas9 technologies offer hope for the development of gene-based therapies for the treatment of LQTS but substantial challenges remain to be overcome.
